# Prognostic marker CD27 and its micro-environmental in multiple myeloma

**DOI:** 10.1186/s12885-024-11945-z

**Published:** 2024-03-19

**Authors:** Xinya Wang, Keyang Luo, Qiuting Xu, Liqun Chi, Yiwei Guo, Chuiming Jia, Lina Quan

**Affiliations:** Hematology Department, Harbin Medical University Cancer Hospital, Harbin, Heilongjiang People’s Republic of China

**Keywords:** CD27, Multiple myeloma, Tumor microenvironment, Prognosis, Treatment

## Abstract

**Background:**

The Cluster of Differentiation 27 (CD27) is aberrantly expressed in multiple myeloma (MM) -derived. This expression facilitates the interaction between tumor and immune cells within TME via the CD27-CD70 pathway, resulting in immune evasion and subsequent tumor progression. The objective of this study is to investigate the correlation between CD27 expression and the prognosis of MM, and to elucidate its potential relationship with the immune microenvironment.

**Methods:**

In this research, CD27 expression in T cells within the 82 newly diagnosed MM microenvironment was assessed via flow cytometry. We then examined the association between CD27 expression levels and patient survival. Subsequent a series of bioinformatics and in vitro experiments were conducted to reveal the role of CD27 in MM.

**Results:**

Clinical evidence suggests that elevated CD27 expression in T cells within the bone marrow serves as a negative prognostic marker for MM survival. Data analysis from the GEO database has demonstrated a strong association between MM-derived CD27 and the immune response, as well as the hematopoietic system. Importantly, patients with elevated levels of CD27 expression were also found to have an increased presence of MDSCs and macrophages in the bone marrow microenvironment. Furthermore, the PERK-ATF4 signaling pathway has been implicated in mediating the effects of CD27 in MM.

**Conclusions:**

We revealed that CD27 expression levels serve as an indicative marker for the prognosis of MM patients. The CD27- PERK-ATF4 is a promising target for the treatment of MM.

**Supplementary Information:**

The online version contains supplementary material available at 10.1186/s12885-024-11945-z.

## Introduction

Multiple myeloma (MM) is a malignancy originating from myeloma clones [1]. Patient survival in MM exhibits significant variability, influenced by genetic factors, patient baseline characteristics, and the bone marrow microenvironment [2]. Current therapeutic strategies focus mainly on supplementing proteasome inhibitors and immunomodulatory drugs with novel agents, leading to a notable enhancement in the overall survival rate of MM patients [3]. However, achieving a clinical cure for MM remains a significant medical challenge. Consequently, there is an imperative need to deepen our understanding of MM’s pathogenesis and to seek more efficacious treatment modalities.

Cluster of Differentiation 27 (CD27), a member of the tumor necrosis factor receptor superfamily, and its natural ligand, CD70, are expressed in T cells, B cells, and NK cells [4]. The interaction between CD27 and CD70 not only triggers T cell proliferation but also stimulates B cell proliferation and plasma cell production [5, 6]. Thus, CD27 emerges as a crucial regulatory molecule within the tumor microenvironment (TME). Recent studies have highlighted that during the transition from monoclonal gammopathy of undetermined significance (MGUS) to MM, there’s a discernible decline in MM-derived CD27 antigen levels [7]. Furthermore, CD27 expression in plasma cells is intricately tied to tumor burden, treatment response, and overall prognosis in MM patients [8]. There’s growing evidence suggesting that drugs targeting CD27 can suppress cell growth and exert antitumor effects through the CD27-CD70 signaling pathway [9]. Yet, the exact mechanism underlying CD27’s role in MM, especially its influence on the bone marrow microenvironment, remains elusive. While the efficacy of CD27-targeting drugs in treating lymphomas and solid tumors has been established in preclinical settings, their potential in MM therapy remains unexplored.

This research investigates the clinical implications of CD27 expression levels in MM and uncover the mechanistic intricacies of CD27’s influence within the bone marrow microenvironment. Our findings underscore that CD27 expression levels can serve as a prognostic indicator for patients. CD27 wields influence on MM tumor cells directly and has a pronounced effect on the MM microenvironment. Its modulatory role appears to be channeled through the activation of the PERK-ATF4 signaling cascade. This research bridges the existing knowledge gap concerning the clinical application and action mechanism of CD27 within the TME, suggesting that CD27-targeted strategies might pave the way for innovative MM treatments.

## Materials and methods

### Patients and sample

A cohort of 82 newly diagnosed MM patients (*n* = 82) admitted to Harbin Medical University Cancer Hospital between January 2016 and October 2020 were included in this study. Diagnostic evaluations encompassed bone marrow assessments, hematological tests, and imaging examinations. The diagnostic criteria set by the International Myeloma Working Group (IMWG) were employed as the foundational guideline for patient diagnoses [10].

### Chemotherapy regimens and detection index

We conducted a retrospective analysis on the 82 MM patients, focusing on age, gender, bone marrow plasma cell (BMPC) proportion, and pretreatment blood indicators. These indicators comprised serum lactate dehydrogenase (LDH), β2-microglobulin (β2-MG), albumin (ALB), serum calcium (Ca), creatinine (Cr), and hemoglobin (Hb).

In line with the NCCN guidelines [11], the 82 confirmed MM patients underwent treatment regimens grounded in proteasome inhibitors. The treatment combinations included VRD (bortezomib + lenalidomide + dexamethasone), PAD (bortezomib + epirubicin + dexamethasone), or PCD (bortezomib + cyclophosphamide + dexamethasone). Utilizing the diagnostic and therapeutic criteria set by the IMWG, treatment efficacy was gauged post the fourth treatment cycle. Treatment responses were categorized as: sCR (stringent complete response), CR (complete remission), VGPR (very good partial remission), PR (partial remission), SD (stable disease), and PD (progressive disease).

### Flow cytometry

Before commencing treatment, bone marrow samples were obtained from the 82 patients using 2 ml of bone marrow fluid. As per the guidelines set forth by the European Myeloma Network (EMN) [12], these samples were stained with specific antibody combinations:CD28-FITC/CD56-PE/CD45-PerCP/CD138-APC; CD20-FITC/CD117-PE/CD45-PerCP/CD138-APC; CD27-FITC/CD28-PE/CD45-PerCP/CD138-APC; and ckappa-FITC/clambda-PE/CD19-PE-Cy7/CD138-APC. All antibodies utilized for the procedure were sourced from BD Bioscience (USA). Subsequent evaluations were conducted to determine the percentage of CD138 + anomalous plasma cells within nucleated cells. Additionally, the expression rate of CD27 in CD3 + T lymphoma cells was ascertained, accompanied by the analysis of other marker expressions. The collected data were analyzed using a FACS Canto II flow cytometer, supplied by BD company, and processed with Diva software. At the point of diagnosis, CD27 expression was classified as positive when it was observed in ≥ 20% of the T-cells.

### Gene Expression Omnibus data source and data processing

Gene expression data and clinical attributes of MM samples were sourced from six independent datasets available on the Gene Expression Omnibus (GEO, http://www.ncbi.nlm.nih.gov/geo/) repository (GSE5900, GSE6477, GSE31161, GSE136337, GSE24080, and GSE57317). Notably, the datasets GSE5900 and GSE6477 were integrated employing the ‘combo’ function within the R sva package. This integration was executed to mitigate batch effects and to standardize the resultant gene expression matrix. The MM diagnosis adhered to the criteria delineated by the World Health Organization (WHO). Additionally, all sample data classifications were grounded in the benchmarks set by the International Myeloma Working Group. The experimental designs, quality assurance, and data normalization processes were all consistent with the established Affimatrix protocol.

### GEO dataset analysis

The datasets GSE5900 and GSE6477 utilize the Affymetrix GPL570 and Affymetrix GPL96 platforms, respectively. Together, these datasets encompass 37 healthy donors, 65 MGUS patients, 35 smoldering multiple myeloma (SMM) patients, and 75 MM patients, resulting in a cumulative 212 samples. We assessed the expression levels of CD27 across these four groups: healthy donors, MGUS, SMM, and MM.

Regarding the Affymetrix GPL570 platform, we extracted gene expression data and associated clinical details from GEO datasets GSE31161, GSE136337, GSE24080, and GSE57317. Specifically: (1) In the GSE31161 dataset, CD27 expression levels were juxtaposed between initially treated patients (*n* = 780) and those experiencing relapse (*n* = 258). (2) For GSE136337, samples from ISS Phases I and II were aggregated (*n* = 303) and juxtaposed against those from ISS Phase III (*n* = 121) to discern disparities in CD27 expression levels. (3) In GSE24080, the primary clinical endpoints were overall survival (OS) spanning 24 months and event-free survival (EFS) also over 24 months. Here, we probed the differential expression of CD27 and ATF4 within two subgroups distinguished by their survival outcomes. (4) Lastly, within GSE57317, samples were bifurcated based on ATF4 gene expression values in the culture medium. We subsequently plotted survival curves to ascertain the overall survival disparities between these expression subgroups.

### Screening and functional enrichment analysis of differentially expressed genes

Utilizing the median CD27 expression from the GSE5900 and GSE6477 datasets as a benchmark, patients with SMM and MM were classified into high and low CD27 expression cohorts. Initial analysis on differential gene expression between these groups was executed with the R limma package. Subsequently, the R ggplot2 package facilitated the creation of a DEGs volcano plot. The R pheatmap package enabled visualization of the leading 15 upregulated genes and the foremost 35 downregulated genes from DEGs, in both high and low expression sample sets. Setting the criteria at | log2FC |> 0.5 and *P* < 0.05, we conducted an enrichment analysis of Gene Ontology (GO), Kyoto Encyclopedia of Genes and Genomes (KEGG), and Gene Set Enrichment Analysis (GSEA) using the R clusterProfiler package. Lastly, the PPI network was mapped using the Search Tool for the Retrieval of Interacting Genes/Proteins (STRING) database, setting *P* < 0.05 as the threshold for meaningful statistical significance.

### Immunocyte infiltration analysis

To assess the matrix of immune cell infiltration, the CIBERSORT algorithm was employed to analyze the gene expression matrix of SMM and MM patients, aiming to ascertain the relative proportions of 22 immune cell types. A criterion of *P* < 0.05 was established for the analysis. Each sample had an aggregated score of 1 from the 22 immune cell types. Leveraging the single-sample gene set enrichment analysis (ssGSEA) method within the R GSVA package, we computed the infiltration magnitude for 28 immune cell types, grounded on gene expression levels within 28 pre-established immune cell gene sets.

### Cells lines and cell culture

MM cell lines, RPMI-8226 and U266, were purchased from ATCC and maintained in RPMI1640 medium (Gibco, Grand Island, NY, USA). This medium was further enriched with 10% fetal bovine serum (FBS, Hyclone, Logan, UT, USA) and 1% penicillin–streptomycin. Cultivation was carried out at 37℃ in a humidity-controlled environment containing 5% carbon dioxide.

### Cell viability and apoptosis

Cell proliferation was evaluated using a CCK8 assay (Solarbio, Beijing, China). Cells were quantified, and 1,000 cells were dispensed into 96-well plates, each well containing 100 μl of medium. They were then treated with CD27 antibody (5 μg/ml, Varlilumab, 1F5, Chemstan, Wuhan, China) and Soluble CD27 (sCD27, 1 ng/ml, ab114342, Abcam, UK) at intervals of 0 h, 48 h, and 72 h. A dimethyl sulfoxide (DMSO, 5ul/ml) treated group served as the control. Subsequently, 10 μl of CCK-8 reagent was introduced to each well, followed by an additional 0.5 h of incubation at 37℃ in a cell incubator (Thermo Forma 311, USA). The absorbance at 450 nm was then ascertained using a microplate reader.

For apoptosis assessment, cells were harvested to yield single-cell suspensions. After undergoing two washes with PBS as the staining buffer, the cells were incubated in the dark at 4℃ for 30 min. They were then stained with Annexin V-FITC and propidium iodide (PI) for 15 min, after which apoptosis was gauged via flow cytometry.

### Validation of cell phenotype

Cells were harvested, washed with FACS buffer, and prepared as suspensions to assess the surface expression of CD27, CD70, and PD-L1 on myeloma cells. The suspensions were then treated with respective antibodies (PD-L1-PE/CD70-PE/ CD27-FITC) sourced from BioLegend, USA, followed by a 15-min incubation. After adding FACS buffer, the samples were centrifuged and the supernatant was removed. These procedures were conducted in light-protected conditions. Subsequent analyses were performed using a CytoFLEX S flow cytometer, and data interpretation was facilitated by the CytExpert software.

### Western blotting

Cells were harvested and subjected to total protein extraction. Protein concentrations were determined using the BCA assay. Proteins were then separated on SDS-PAGE gels, after which the samples were boiled for 3–5 min. The separated proteins were transferred to PVDF membranes (Millipore, USA). After blocking with 5% skim milk for an hour, the membranes were incubated overnight at 4℃ with specific primary antibodies. Following overnight incubation, the membranes were washed with TBST and any unbound antibodies were removed. Subsequently, membranes were incubated at 37℃ for 2 h with horseradish peroxidase-conjugated secondary antibodies. Visualization of the proteins was achieved using a chemiluminescence detection system.

### QRT-PCR analysis

Total RNA was extracted from RPMI-8226 and U266 tumor cells utilizing the Trizol reagent (Invitrogen, Carlsbad, CA, USA). The RNA’s integrity and concentration were assessed using the SmartSpec Plus Spectrophotometer (Bio-Rad Laboratories, Inc., Hercules, CA, USA). The reverse transcription process was executed using the ReverTra Ace qPCR RT Kit (TOYOBO Life Science, Shanghai, China) as per the provided manufacturer’s protocol. Quantitative real-time PCR (qRT-PCR) was employed to evaluate the expression levels of protein kinase R (PKR)-like endoplasmic reticulum (ER) kinase (PERK) and activating transcription factor 4 (ATF4) genes. This qRT-PCR assay was conducted on a Bio-Rad S1000 thermal cycler using Bestar SYBR Green RT-PCR Master Mix (TOYOBO). Relative gene expression quantification was determined using the 2–ΔΔCt approach, as described by Livak and Schmittgen (2001). Each sample underwent PCR amplifications in triplicate. The specific primer sequences utilized are listed in Table S1.

### Statistical analysis

SPSS 26.0 and GraphPad Prism software were used for statistical analysis and processing of data, and T-test or one-way analysis of variance was used for the difference between groups. Data are expressed as mean ± SD. Kaplan–meier method was used for survival analysis, and Log-rank test was used for survival analysis. COX proportional hazard model was used for univariate and multivariate analysis. All experiments were repeated three times. *P* < 0.05 was considered statistically significant.

## Results

### CD27 expression and patient clinical characteristics

Based on the expression levels of CD27 + T lymphocytes in MM cells, MM patients were categorized into two groups: the CD27 + group (≥ 20%) and the CD27- group (< 20%). The association between CD27 expression and various clinical features was analyzed and presented in Table 1. Notably, the CD27 + group exhibited elevated levels of β2-MG (65.00% vs. 36.36%, *P* = 0.020) and BMPC proportion (60.00% vs. 27.27%, *P* = 0.009) in comparison to the CD27- group. Furthermore, the proportion of patients in stage III of the International Staging System (ISS) was considerably lower in the CD27- group than in the CD27 + group (22.73% vs. 33.33%, *P* < 0.001). However, factors such as gender, age, Hb, Ca, ALB, Cr, and LDH did not exhibit significant variations between the two groups (*P* > 0.05).

**Table 1:** Correlation analysis between CD27 expression and clinical factors in 82 patients

Variable	Subcategory	CD27 + group (*n* = 60)	CD27-group (*n* = 22)	*P* value
Gender	female	27(45.00%)	10(45.45%)	0.971
	male	33(55.00%)	12(54.55%)	
Age(year)	≥ 65	25(41.67%)	12(54.55%)	0.299
	< 65	35(58.33%)	10(45.45%)	
HGB(g/L)	≥ 100	38(63.33%)	10(45.45%)	0.145
	< 100	22(36.67%)	12(54.55%)	
Ca(mmol/L)	≥ 2.75	12(20.00%)	5(22.73%)	0.787
	< 2.75	48(80.00%)	17(77.28%)	
ALB(g/L)	≥ 35	31(51.67%)	15(68.18%)	0.182
	< 35	29(48.33%)	7(31.82%)	
Cr(μmol/L)	≥ 177	18(30.00%)	8(36.36%)	0.583
	< 177	42(70.00%)	14(63.64%)	
LDH(U/L)	≥ 246	24(40.00%)	12(54.55%)	0.240
	< 246	36(60.00%)	10(45.45%)	
β2-MG (mg/L)	≥ 3.5	39(65.00%)	8(36.36%)	0.020
	< 3.5	21(35.00%)	14(63.64%)	
BMPC proportion (%)	≥ 31.49	36(60.00%)	6(27.27%)	0.009
	< 31.49	24(40.00%)	16(72.73%)	
ISS stage	I -II	40(66.67%)	17(77.28%)	< 0.001
	III	20(33.33%)	5(22.73%)	

*BMPC* bone marrow plasma cell, *LDH* lactate dehydrogenase, *β2-MG* β2-microglobulin, *ALB* albumin, *Ca* serum calcium, *Cr* creatinine, *Hb* hemoglobin, *ISS* International Staging System

### Differential expression of CD27 and treatment response

All of the enrolled 82 MM patients completed four cycles of bortezomib-based induction therapy. Treatment strategies were bifurcated into either immunomodulator-based or chemotherapy-based, and no significant efficacy difference was observed between them. Efficacy assessment, based on IMWG criteria, revealed: 1.22% achieved a stringent complete response (sCR), 10.98% a complete response (CR), 19.51% a very good partial response (VGPR), 30.49% a partial response (PR), 21.95% had stable disease (SD), and 15.85% experienced progressive disease (PD). Notably, the CD27- group manifested a superior overall response rate (ORR sum of sCR, CR, VGPR, and PR) at 81.82% compared to 55.00% in the CD27 + group. Deep remission (encompassing sCR, CR, and VGPR) was observed in 12 cases for the CD27- group and 14 for the CD27 + group, marking a significant divergence between them. Further, the CD27 + group registered a higher ineffective rate (combining SD and PD) than the CD27- group, with percentages standing at 45.00% and 18.18% respectively, as delineated in Table 2.

**Table 2:** Correlation analysis between CD27 expression level and short-term efficacy

Characteristic	CD27 + group (*n* = 60)	CD27-group (*n* = 22)	*P* value
Immunomodulatory -based regime (VRD)	22(36.67%)	12(54.55%)	0.145
Chemotherapy-based regime (PCD/PAD)	38(63.33%)	10(45.45%)	0.145
sCR	0(0.00%)	1(4.55%)	0.097
CR	4(6.67%)	5(22.73%)	0.039
VGPR	10(16.67%)	6(27.27%)	0.283
PR	19(31.67%)	6(27.27%)	0.702
SD	16(26.67%)	2(9.09%)	0.088
PD	11(18.33%)	2(9.09%)	0.310
ORR	33(55.00%)	18(81.82%)	0.026
sCR + CR + VGPR	14(23.33%)	12(54.55%)	0.007
SD + PD	27(45.00%)	4(18.18%)	0.026

VRD bortezomib + lenalidomide + dexamethasone, PAD bortezomib + epirubicin + dexamethasone, PCD bortezomib + cyclophosphamide + dexamethasone, *sCR* stringent complete response, *CR* complete remission, *VGPR* very good partial remission, *PR* partial remission, *SD* stable disease, *PD* progressive disease, ORR sum of sCR, CR, VGPR, and PR

### Effect on long-term survival in two groups of MM patients

Until October 2020, the median follow-up time of 82 MM patients was 16 months. The median progression-free survival (PFS) and OS of the CD27 + group were shorter than those of the CD27-group (13 vs. 24 months, *P* = 0.02, and both OS not reached, *P* = 0.002) (Fig. S1A and B). Univariate analysis showed that ISS stage III, CD27 + T cell ≥ 20%, BMPC proportion ≥ 31.49%, age ≥ 65 years, β_2_-MG ≥ 3.5 mg/L, affected the PFS of MM patients (*P* < 0.05). ISS stage III, CD27 + T cell ≥ 20%, BMPC proportion ≥ 31.49%, age ≥ 65 years were related to the OS of MM patients (*P* < 0.05). Other detection indicators were not risk factors for PFS and OS (*P* > 0.05) (Fig. S1C and D). After excluding the interaction of each factor, a significant single factor Cox multivariate analysis showed that CD27 + T cell ≥ 20% was an independent prognostic factor affecting progression-free survival and overall survival of MM patients (Tables 3 and 4).

**Table 3:** Risk factors affecting PFS in MM patients

Risk factor	Univariate analysis HR 95%CI *P* value	Multivariate analysis HR 95%CI *P* value
ISS stage III	1.957 1.094–3.500 0.024	0.873 0.387–1.971 0.744
Cr ≥ 115 μmol/L	1.031 0.589–1.805 0.915	
CD27 + T ≥ 20%	4.147 2.015–8.535 0.000	3.759 1.730–8.168 0.001
BMPC ≥ 31.49%	1.807 1.054–3.099 0.032	1.143 0.630–2.075 0.659
Ca ≥ 2.75mmlo/L	0.891 0.459–1.729 0.733	
HB < 100 g/L	0.726 0.420–1.254 0.251	
LDH > 246U/L	0.712 0.420–1.209 0.209	
ALB < 35 g/L	1.402 0.818–2.402 0.219	
Age ≥ 65	1.940 1.128–3.338 0.017	2.068 1.068–4.003 0.031
β2-MG ≥ 3.5 μmol/L	2.556 1.431–4.565 0.002	1.433 0.689–2.981 0.336
Therapeutic schemes	0.662 0.388–1.130 0.131	

*HR* hazard ratio, *CI* confidence interval

**Table 4:** Risk factors affecting OS in MM patients

Risk factor	Univariate analysis HR 95%CI *P* value	Multivariate analysis HR 95%CI *P* value
ISS stage III	2.048 1.003–4.182 0.049	1.046 0.438–2.501 0.919
Cr ≥ 115 μmol/L	0.934 0.468–1.866 0.848	
CD27 + T ≥ 20%	3.542 1.487–8.436 0.004	3.353 1.322–8.504 0.011
BMPC ≥ 31.49%	2.214 1.141–4.297 0.019	1.501 0.729–3.090 0.270
Ca ≥ 2.75mmlo/L	0.918 0.398–2.119 0.842	
HB < 100 g/L	1.561 0.280–1.123 0.103	
LDH > 246U/L	0.770 0.401–1.480 0.434	
ALB < 35 g/L	1.903 0.973–3.722 0.060	
Age ≥ 65	2.336 1.186–4.601 0.014	2.426 1.038–5.670 0.041
β2-MG ≥ 3.5 μmol/L	1.889 0.943–3.783 0.073	
Therapeutic schemes	1.617 0.316–1.205 0.158	

*HR* hazard ratio, *CI* confidence interval

### Prognostic value of CD27 expression in MM

**Fig. 1:**
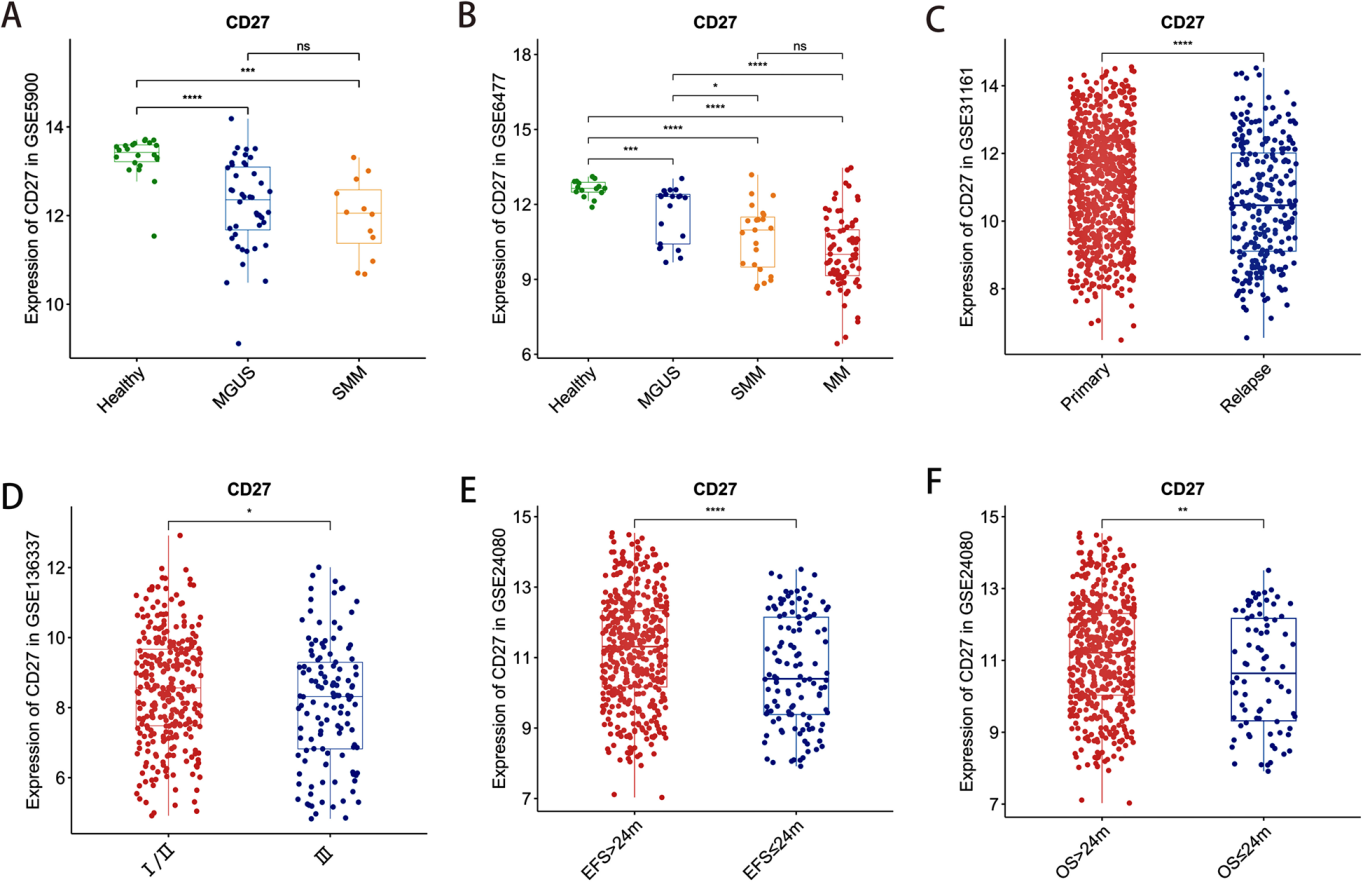
The expression level of CD27 in MM patients with different clinical characteristics. **A**, **B** CD27 expression level in different subtypes of myeloma patients in GSE5900 and GSE6477. **C** The expression level of CD27 in different recurrence states of MM in GSE31161. **D** Comparison of CD27 expression levels between ISS I/II and ISS III phases in GSE136337. **E**, **F** Expression of CD27 in different survival subgroups (EFS24m and OS24m) in GSE24080. * *P* < 0.05, ** *P* < 0.01, *** *P* < 0.001 and **** *P* < 0.0001

In MM, malignant plasma cells are the primary pathogenic entities. A study conducted at a single center revealed a correlation between CD27 expression in plasma cells and the prognosis of MM patients [8]. To extend these findings, we conducted a comprehensive analysis of the prognostic significance of CD27 in plasma cells using various databases. The observations highlighted a discernible decline in CD27 expression levels concomitant with the progression of myeloma, corroborating the outcomes derived from flow cytometry [7]. Specifically, in the GSE5900 dataset, we identified a marked decrease in CD27 expression when comparing Normal (*n* = 22), MGUS (*n* = 44), and SMM (*n* = 12) cases (*P* values being 1.1e-07, 0.00017, and 0.35, respectively, as illustrated in Fig. 1A). This trend was further confirmed in the GSE6477 dataset, where a consistent decline in CD27 expression was observed across different stages, namely Normal (*n* = 15), MGUS (*n* = 22), SMM (*n* = 24), and MM (*n* = 101) (with *P* values of 0.00062, 3.2e-07, 2.22e-16, 0.012, 5.3e-06, and 0.089, respectively, depicted in Fig. 1B). In conclusion, the diminishing expression of CD27 in monoclonal gammopathy underscores the potential role of CD27 in the malignant evolution of myeloma.

To further explore the value of CD27 expression differences on the survival of MM, we evaluated its expression under various clinical scenarios, i.e., different relapse statuses, different ISS stages, and survival times in MM patients. In GSE31161 dataset, we observed that the expression levels of CD27 in the primary group (*n* = 780) were higher than in the relapsed group (*n* = 255) (*P* < 0.0001, Fig. 1C). We also compared the expression of CD27 in different ISS stage. In GSE136337, ISS stages I and II were combined into one group for comparison with ISS stage III. we noticed a significant increase of CD27 expression in the ISS I and II phase of MM (*n* = 303) compared to the ISS III phase of MM (*n* = 121) (*P* = 0.023, Fig. 1D). In GSE24080, compared with the EFS > 24 months subgroup (*n* = 441), the EFS ≤ 24 months subgroup (*n* = 118) had lower expression of CD27 (*P* < 0.0001, Fig. 1E); Compared with the OS > 24 months subgroup (*n* = 481), the expression of CD27 was lower in the OS ≤ 24 months subgroup (*n* = 78) (*P* < 0.0087, Fig. 1F). The above results indicated the expression level of CD27 may be one of the indicators to MM prognosis.

### CD27 pathway analysis and PPI Network in MM

**Fig. 2:**
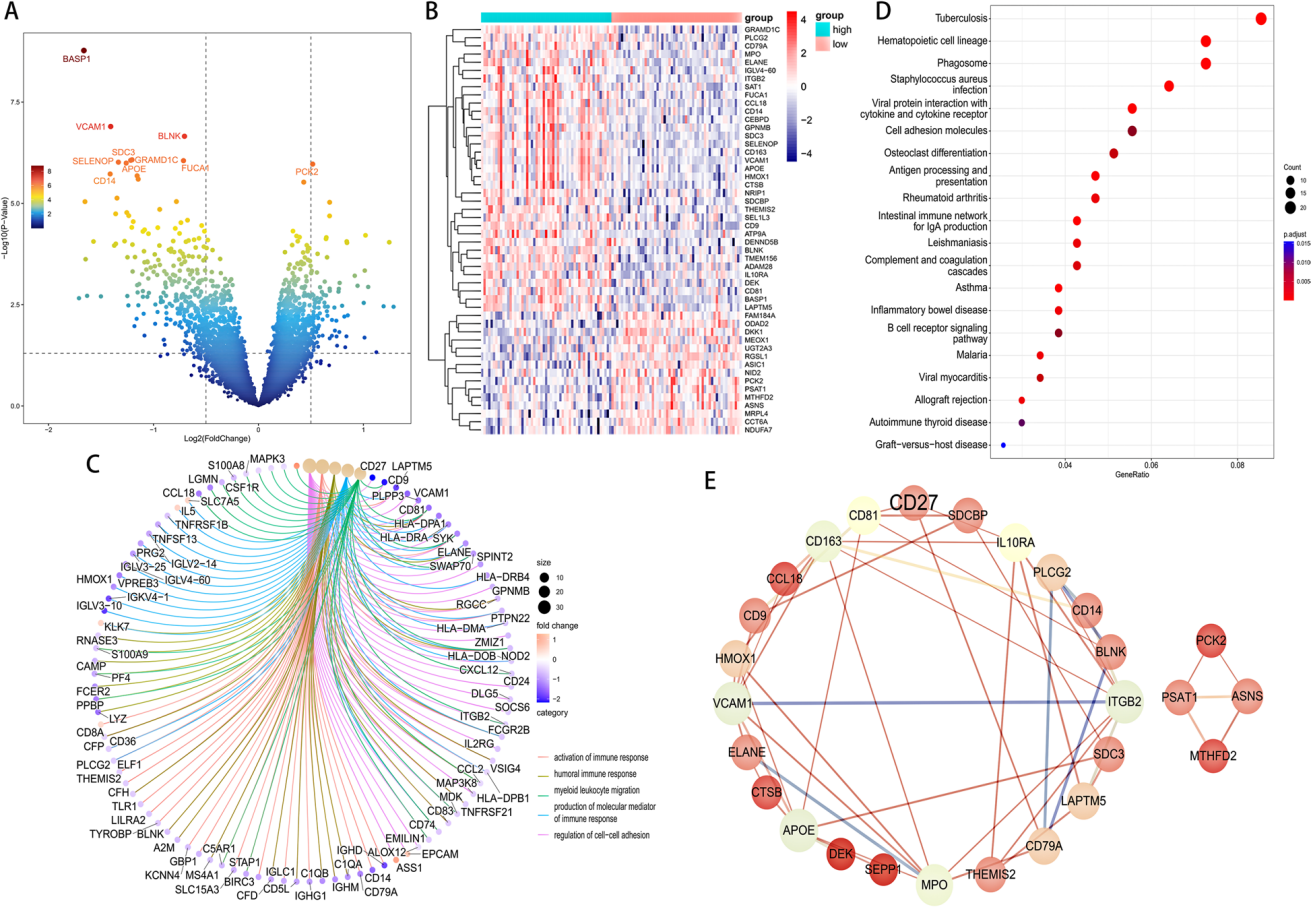
Enrichment analysis and PPI network of CD27 differentially expressed genes in MM. **A** Volcano map of CD27 differentially expressed genes in the GSE5900 and GSE6477. **B** Heatmap of the 50 differentially expressed genes. The color from Red to blue represents that the genes are from high to low. **C** GO enrichment analysis of DEGs. D KEGG enrichment analysis of DEGs. E PPI network of CD27

To examine the functional roles of the MM-derived CD27, we employed differential gene expression analysis using the fold change methodology. Utilizing the median expression value of CD27 from the amalgamated GSE5900 and GSE6477 datasets, we stratified SMM and MM patients into high and low expression cohorts. This partitioning led to the identification of 101 upregulated and 326 downregulated genes (Fig. 2A). A heat map showed the predominant 15 upregulated and 35 downregulated genes (Fig. 2B). Next, we analyzed GO terms and KEGG pathways associated with CD27. GO term enrichment analysis of these DEGs revealed that they were contacted with the activation of immune responses, humoral immune responses, myeloid leukocyte migration, production of molecular mediators of immune response and regulation of cell–cell adhesion signaling (Fig. 2C). KEGG pathway analysis further associated these DEGs with distinct processes such as tuberculosis, hematopoietic cell lineage, phagosome formation, and staphylococcus aureus infection pathways (Fig. 2D).

To elucidate the potential protein interactions of these DEGs, we employed the Cytoscape software to generate a protein–protein interaction (PPI) network. This was achieved using the String database, encompassing the top 15 upregulated and 35 downregulated genes. The resulting PPI network revealed that of the 35 downregulated genes, 21 (including CD163, ITGB2, VCAM1, and CCL18) showcased interaction relationships. In contrast, among the 15 upregulated genes, 4 (comprising PCK2, ASNS, PSAT1, and MTHFD2) were interactive (Fig. 2E). Crucially, CD27 emerged as a central node in the overarching network, predominantly among the downregulated genes. Collectively, both the pathway enrichment and PPI network analyses underscore the potentially pivotal role of CD27 in the pathophysiology of MM.

### CD27 expression is linked to the MM tumor microenvironment

**Fig. 3:**
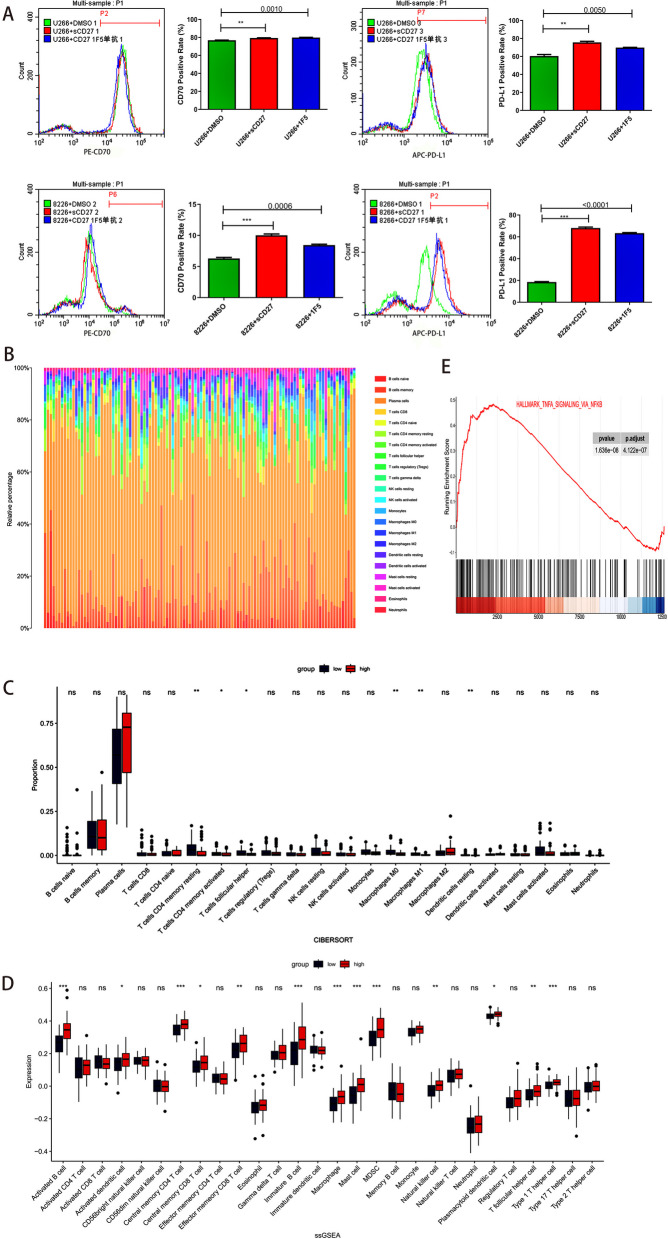
Correlation analysis of the microenvironment. **A** Flow cytometry detection of CD70 and PD-L1 expression in U266 and RPMI-8226 MM cells treated with sCD27 and 1F5. **B** Bar plot showing the ratio of 22 immune cell types in GSE5900 and GSE6477 samples. **C** Box line plots are used to represent the 22 immune cell types in the low and high CD27 patient cohorts in blue and red, respectively. **D** Comparison of ssGSEA for representation of 28 immune cell expressions in low and high CD27 patient cohorts. **E** Significantly enriched NK-κ B signaling pathway in GSEA. * *P* < 0.05, ** *P* < 0.01, *** *P* < 0.001 and **** *P* < 0.0001

CD27, a prominent protein in the tumor microenvironment, modulates cellular activity by engaging with CD70. Our flow cytometry analysis illuminated that CD27 markedly augmented the expression levels of both CD70 and PD-L1 relative to the DMSO control group (Fig. 3A). In congruence with prior research indicating that heightened PD-L1 expression is integral to the tumor microenvironment [13], we delved deeper into the composition of immune infiltrates in myeloma and its interplay with CD27 expression. Utilizing the CIBERSORT algorithm, we charted the distribution of 22 tumor-infiltrating immune cell (TIIC) phenotypes in MM (Fig. 3B). Intriguingly, our analysis unveiled that, relative to their CD27-high counterparts, patients with low CD27 expression manifested an elevated proportion of CD4 + resting memory T cells and M1 macrophages, and a diminished fraction of dendritic cells (*P* < 0.01) (Fig. 3C). Subsequent ssGSEA exploration highlighted that 13 immune cell subgroups-including macrophages, myeloid-derived suppressor cells, and Th1 cells—were more abundant in the CD27-high cohort compared to the CD27-low group (*P* < 0.001) (Fig. 3D). GSEA further suggested that CD27 might influence the myeloma microenvironment via the nuclear factor-κB (NF-κB) signaling cascade (Fig. 3E). Collectively, these findings underscore the intricate relationship between CD27 and TME.

### PERK-ATF4 signaling pathway is involved in CD27’s effect in MM

**Fig. 4:**
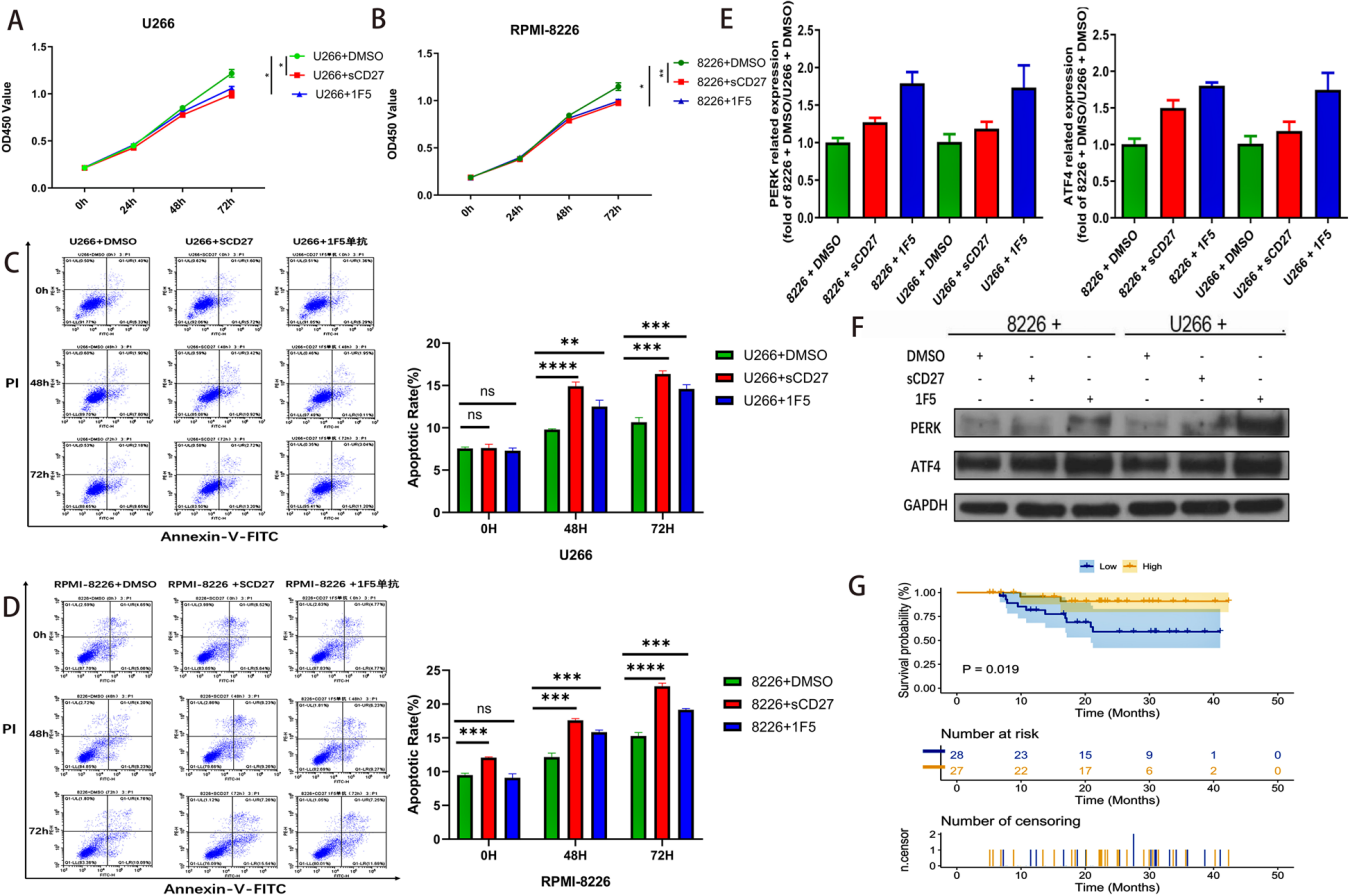
The effect of CD27 on proliferation and apoptosis of myeloma cells. **A**, **B** CCK-8 was used to detect the proliferation of U266 and RPMI-8226 cells treated with sCD27 and 1F5 for 0, 24, 48, and 72 h. **C**, **D** Flow cytometry was used to analyze the apoptosis of U266 and RPMI-8226 cells treated with sCD27 and 1F5 for 0, 48, and 72 h. **E** qPCR detection of mRNA levels of PERK and ATF4 in MM cells treated with sCD27 and 1F5. **F** Western blotting detection of protein levels of PERK and ATF4 in MM cells treated with sCD27 and 1F5. **G** The correlation between ATF4 expression and OS of MM patients in GSE57317 was conducted by Kaplan–Meier analysis. * *P* < 0.05, ** *P* < 0.01, *** *P* < 0.001 and **** *P* < 0.0001

To elucidate the impact of CD27 expression on MM cell behavior, we examined the implications of enhancing CD27 expression on MM proliferation. In the MM cell lines U266 and RPMI-8226, treatment with CD27 stimulators (sCD27 and 1F5) across 0 h, 24 h, 48 h, and 72 h intervals manifested a notable reduction in cell viability, especially with DMSO as a control, highlighting a time-dependent inhibition of MM cell growth (Fig. 4A, B). Enhanced CD27 expression also accelerated MM cell apoptosis, with CD27 stimulators inducing apoptosis progressively (*P* < 0.05) (Fig. 4C, D). The above data suggested that expression of CD27 could inhibit the growth of myeloma cells and promote the apoptosis of myeloma cells. The activation of the pro-apoptotic pathway in myeloma cells was often caused by unfolded protein response (UPR). One of the classics among UPR pathways is mediated by PERK-ATF4 [14]. As expected, compared with the DMSO group, CD27 expression was increased in myeloma cells, PERK and ATF4 mRNA and protein expression were correspondingly increased (Fig. 4E, F). Besides, we found that the ATF4 low subgroup had significantly poorer survival, including both OS and EFS, compared to the ATF4 high subgroup in two independent datasets of GSE57317 and GSE240080 (Fig. 4G; Fig. S2). In conclusion, CD27 may affect myeloma cell activity through the PERK-ATF4 signaling pathway, which in turn affected the survival of MM patients.

## Discussion

MM is characterized as a malignancy resulting from unchecked plasma cell proliferation. CD27, whose expression in MM varies, has been linked to patient outcomes; specifically, diminished CD27 expression correlates with a heightened tumor burden and suboptimal therapeutic responses [15–17]. While prior research has emphasized the clinical significance of CD27 in plasma cells of MM patients, its potential role within myeloma’s microenvironmental T cells and its broader mechanistic implications in MM remain uncharted territories. Our findings spotlight CD27 as a prognostic indicator for MM, deeply entrenched in the immunoregulatory and hematopoietic pathways inherent to the disease. The CD27-CD70 axis emerges as pivotal in MM pathogenesis, with CD27 modulating elements within the myeloma microenvironment, thus impacting patient prognosis. Furthermore, CD27 demonstrates the capability to suppress MM cell proliferation and instigate apoptosis via the PERK-ATF4 signaling cascade. Given these revelations, CD27 exploration holds promise, potentially unveiling a novel biomarker and therapeutic target for MM.

T cells and the cellular immune phenotype within the bone marrow microenvironment play a pivotal role in determining the prognosis of MM patients [18]. CD27, a transmembrane phosphoglycoprotein present on T cells, engages with CD70, facilitating the formation and activation of effector T cells [19]. Up until now, literature has been devoid of studies investigating the implications of CD27 expression on T lymphocytes in the bone marrow microenvironment concerning MM patient prognosis. Our research endeavors to bridge this knowledge gap (Table S2). Our current findings indicate that the median CD27 expression on T lymphocytes within the MM patient bone marrow microenvironment stands at 32.50% (ranging from 0.40% to 65.40%). We observed distinct disparities in tumor burden and treatment outcomes between the CD27- and CD27 + cohorts. Specifically, advanced-stage patients in the CD27 + group manifested increased tumor burdens and diminished therapeutic efficacy. Conversely, the CD27- group displayed enhanced PFS and OS metrics. Notably, a CD27 expression in bone marrow T cells at or above 20% was identified as an independent poor prognostic factor for MM patients. These revelations underscore the potential of CD27 as a diagnostic marker for delineating the clinical trajectory and prognostication of MM.

MM often originates from asymptomatic precursor conditions such as MGUS or SMM. Throughout the disease’s progression or following treatment, CD27 expression in MM patients exhibits notable alterations [17]. Research indicates that a decline in CD27 expression in plasma cells is directly associated with the advancement of MM [7]. A study by Chu et al. [8] highlighted that patients who exhibited a lower expression of CD27 in their plasma cells faced considerably reduced PFS and OS durations. These findings align with data sourced from the GEO database in our study, corroborating that diminished CD27 expression in myeloma plasma cells is indicative of an adverse prognosis.

CD27 plays a pivotal role in numerous immune responses, acting as a co-stimulator for human T cell activities [20]. Under inflammatory conditions, the CD27-CD70 interaction governs the differentiation process of white blood cells via negative feedback, subsequently influencing hematopoietic function [21]. These insights provide a rationale for the pronounced enrichment of immune response and hematopoietic system pathways observed in our research. Proteins interacting with MM-derived CD27, including VCAM-1, CCL18, ITGB2s, HMOX1, and APOE, have also been linked with MM or hematopoietic cell pathways as identified in the PPI network [22–25]. Collectively, our findings underscore the significance of the CD27-CD70 axis in MM, suggesting a notable association between CD27 expression and MM patient prognosis.

Within the TME, the CD27-CD70 interaction spurs the activity of immune cells through the NF-κ B signaling pathway, fostering the phenomenon of immune escape [4, 22]. Notably, the PD-1/PD-L1 signaling pathway stands out as a quintessential mechanism for tumor immune evasion, with PD-L1 expression being modulated by elements within the tumor microenvironment [13]. In line with this, our findings corroborate the notion that CD27-CD70 potentially influences the myeloma microenvironment via the NF-κ B and PD-L1 signaling pathways. Furthermore, we discerned that CD27 steers the accumulation of MDSCs and macrophages. Both MDSCs and macrophages are documented to promote tumor cell proliferation and immunosuppression by reshaping the myeloma microenvironment [26, 27]. This leads to the inference that the adverse prognosis observed in patients with heightened CD27 expression in MM could be attributed to an augmented presence of myeloid-derived suppressor cells and macrophages in the myeloma microenvironment. In essence, our data accentuates the intrinsic link between CD27 expression and the dynamics of the TME.

MM characteristically exhibits an active proteasome pathway. When this pathway is hindered, there is an abnormal deposition of misfolded proteins within the endoplasmic reticulum (ER), thereby triggering an ER stress response [1]. PERK functions as a critical stress sensor within the ER, initiating an ER stress response through ATF4 activation, ultimately leading to apoptosis in MM cells [14]. Our in vitro studies have demonstrated that CD27 limits MM cell proliferation and promotes apoptosis, with the PERK-ATF4 pathway playing a significant role in CD27’s impact on MM. Intriguingly, MM patients with increased ATF4 expression exhibited longer survival periods, suggesting a synergistic effect between CD27 and ATF4. The improved survival in patients expressing both CD27 and ATF4 at high levels might be attributable to the PERK-ATF4 pathway. Furthermore, our experiments with the CD27-targeted therapeutic agent, 1F5, revealed its efficacy in inhibiting MM cell growth. Given the recent successes of immunotherapy in MM treatment [28–30], combining CD27 targeting with immunotherapy to modulate T and NK cell activity in the tumor microenvironment appears to be a promising approach.

This study highlights CD27’s critical role as a diagnostic and prognostic biomarker in MM. The impact of CD27 on MM patient prognosis varies across different cell types, possibly because CD27-T cells mainly participate in CD8 + memory T cell activities, influencing prognosis through cellular immune responses [31, 32]. In contrast, plasma cell CD27 + may be involved in cell apoptosis pathways. Our findings reveal that MM-derived CD27 orchestrates immune-related pathways and the hematopoietic system, thereby facilitating MM progression. The CD27-CD70 axis is identified as a key signaling pathway in MM, regulating the cellular composition of the bone marrow microenvironment. Increased CD27 expression also inhibits MM cell proliferation and encourages apoptosis. The involvement of the PERK-ATF4 pathway in CD27’s role in MM is notable. In conclusion, CD27 not only offers potential clinical value for hematological malignancies but also sets the stage for further detailed studies into the mechanisms underlying CD27’s functions in MM.

### Supplementary Information

**Additional file 1: Figure S1.** Analyzing the risk factors affecting the prognosis of MM patients.

**Additional file 2: Figure S2.** Expression of ATF4 in different survival subgroups (EFS24m) in GSE24080.

**Additional file 3: Table S1.** Designed primers for RT-qPCR.

**Additional file 4: Table S2.** Basic clinical characteristics of 82 MM patients.

**Additional file 5.**

## Data Availability

All the data corresponding to the MM series used in this study are available in GEO (https://www.ncbi.nlm.nih.gov/geo), which are public functional genomics data repositories. The data analyzed during this study are included in the published article and supplementary materials. No more additional data is generated.
