# Supplementary material for: Prognostic marker CD27 and its micro-environmental in multiple myeloma
Source: BMC Cancer. 2024 Mar 19;24:352. doi: 10.1186/s12885-024-11945-z (PMC10949675; doi:10.1186/s12885-024-11945-z)
Supplement: Supplementary file 1 — Additional file 1: Figure S1. Analyzing the risk factors affecting the prognosis of MM patients. [file 12885_2024_11945_MOESM1_ESM.pdf]

FigureS1

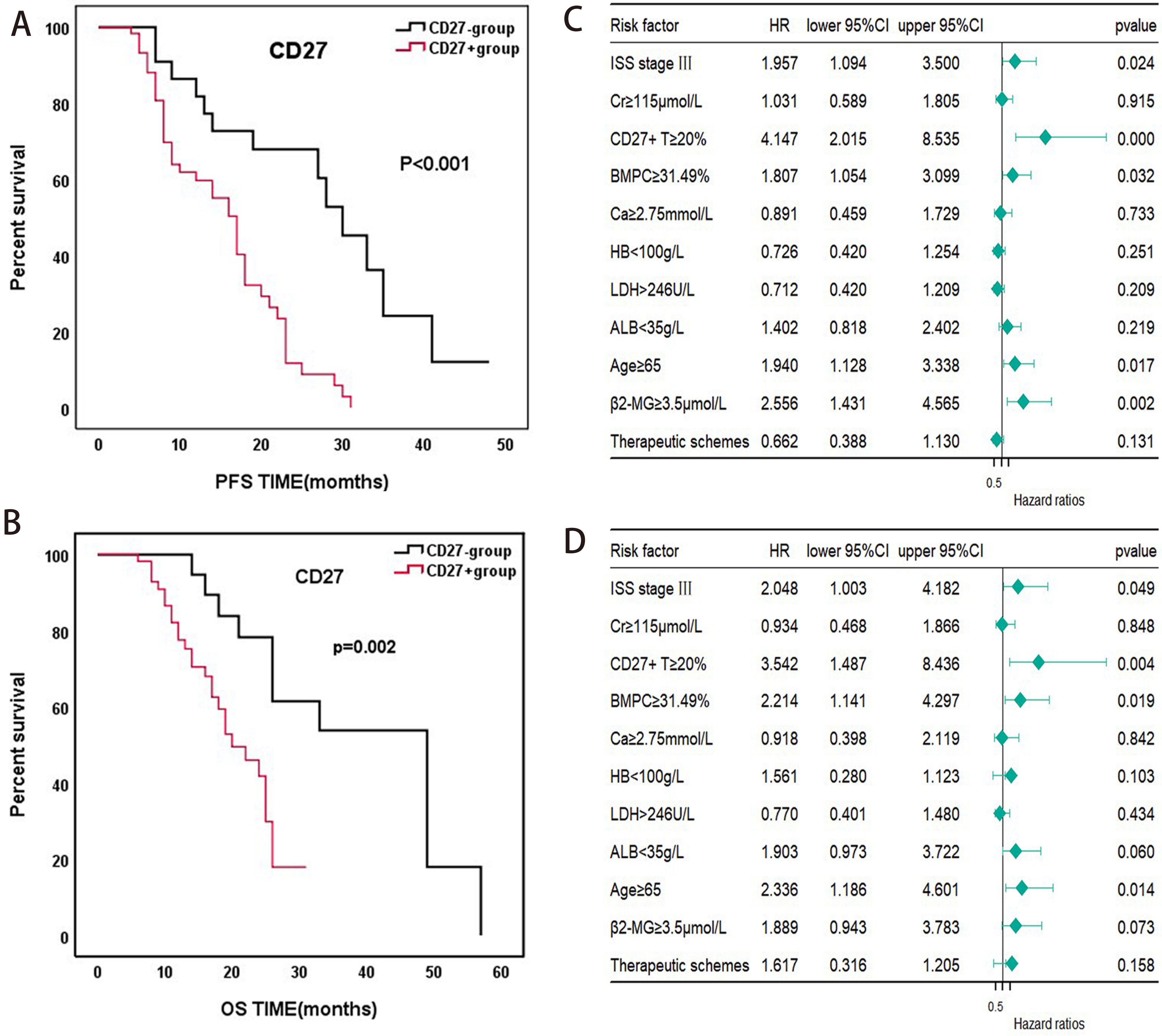

Figure S1. Analyzing the risk factors affecting the prognosis of MM patients. A Kaplan- Meier survival analysis of the correlation between CD27 expression level and PFS in MM patients. B Kaplan-Meier survival analysis of the correlation between CD27 expression level and OS in MM patients. C The forest map shows the correlation between each factor and the prognosis of PFS in MM patients through COX single factor analysis. For each specific influencing factor, it is represented by a vertical line, and the horizontal line crossing the vertical line represents a 95% confidence interval. D The forest map shows the correlation between each factor and the prognosis of OS in MM patients through COX single factor analysis.
