# Supplementary figures and images for: Prognostic marker CD27 and its micro-environmental in multiple myeloma
Source: BMC Cancer. 2024 Mar 19;24:352. doi: 10.1186/s12885-024-11945-z (PMC10949675; doi:10.1186/s12885-024-11945-z)

FigureS2

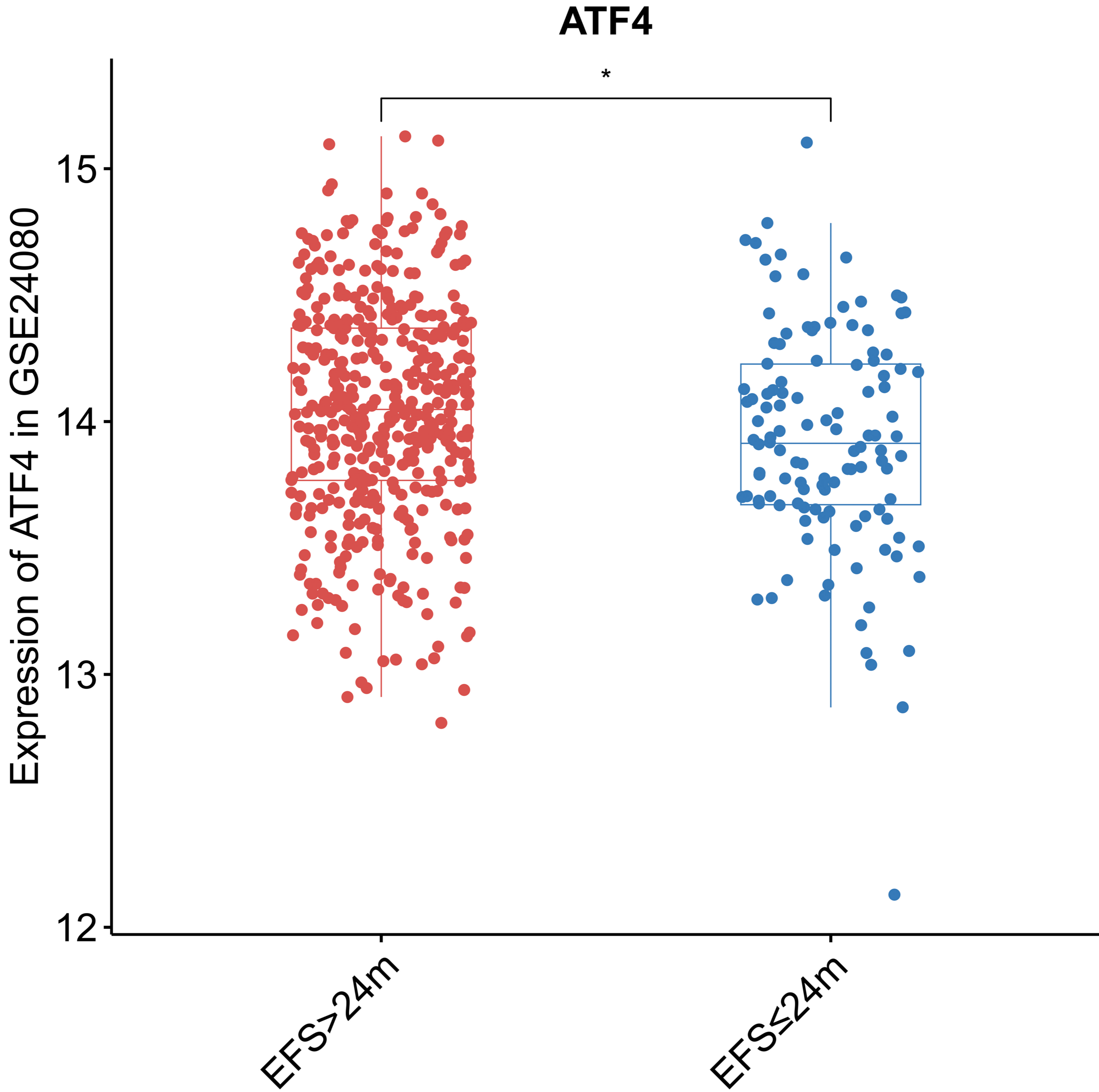

Figure S2 Expression of ATF4 in different survival subgroups (EFS24m) in GSE24080.

Supplement: Supplementary file 2 — Additional file 2: Figure S2. Expression of ATF4 in different survival subgroups (EFS24m) in GSE24080. [file 12885_2024_11945_MOESM2_ESM.pdf]

Figure4F

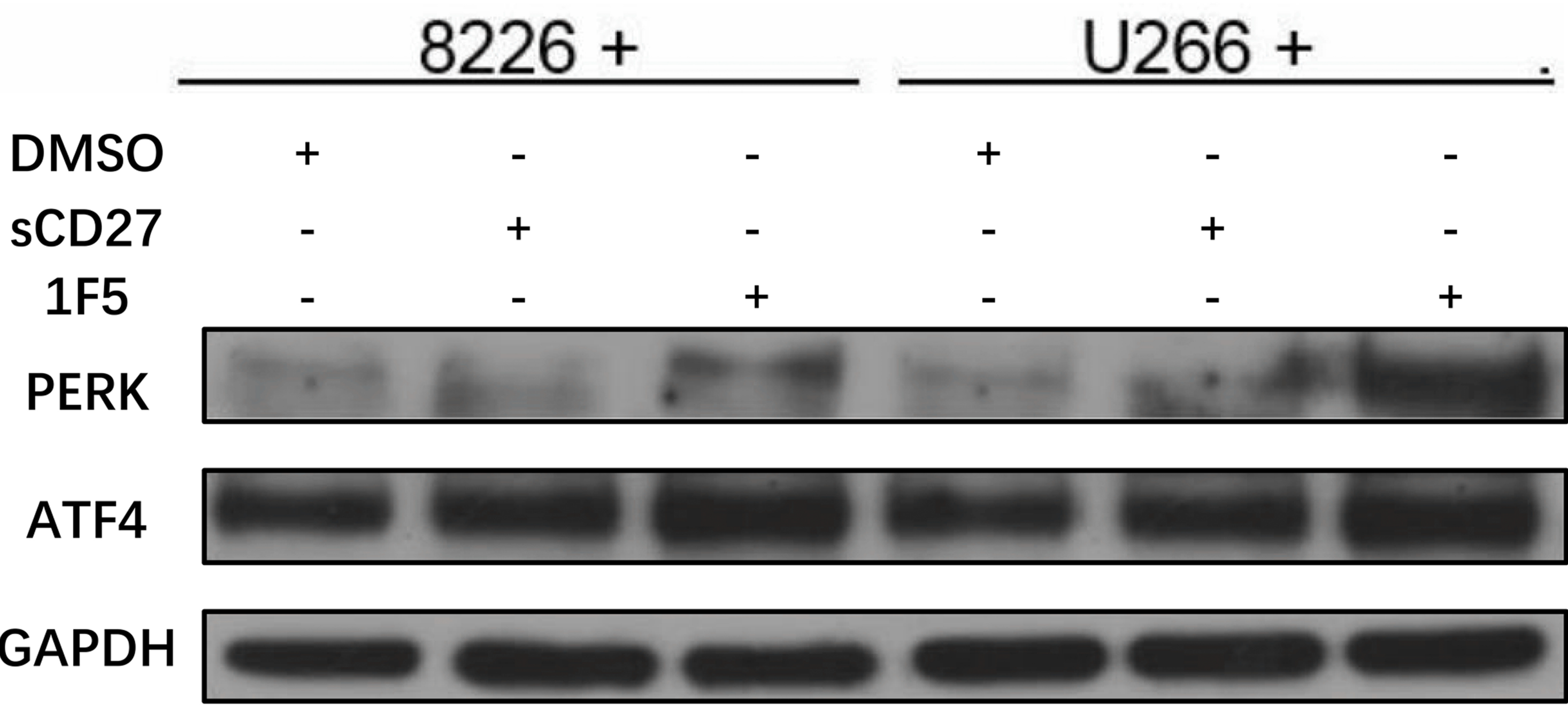

PERK

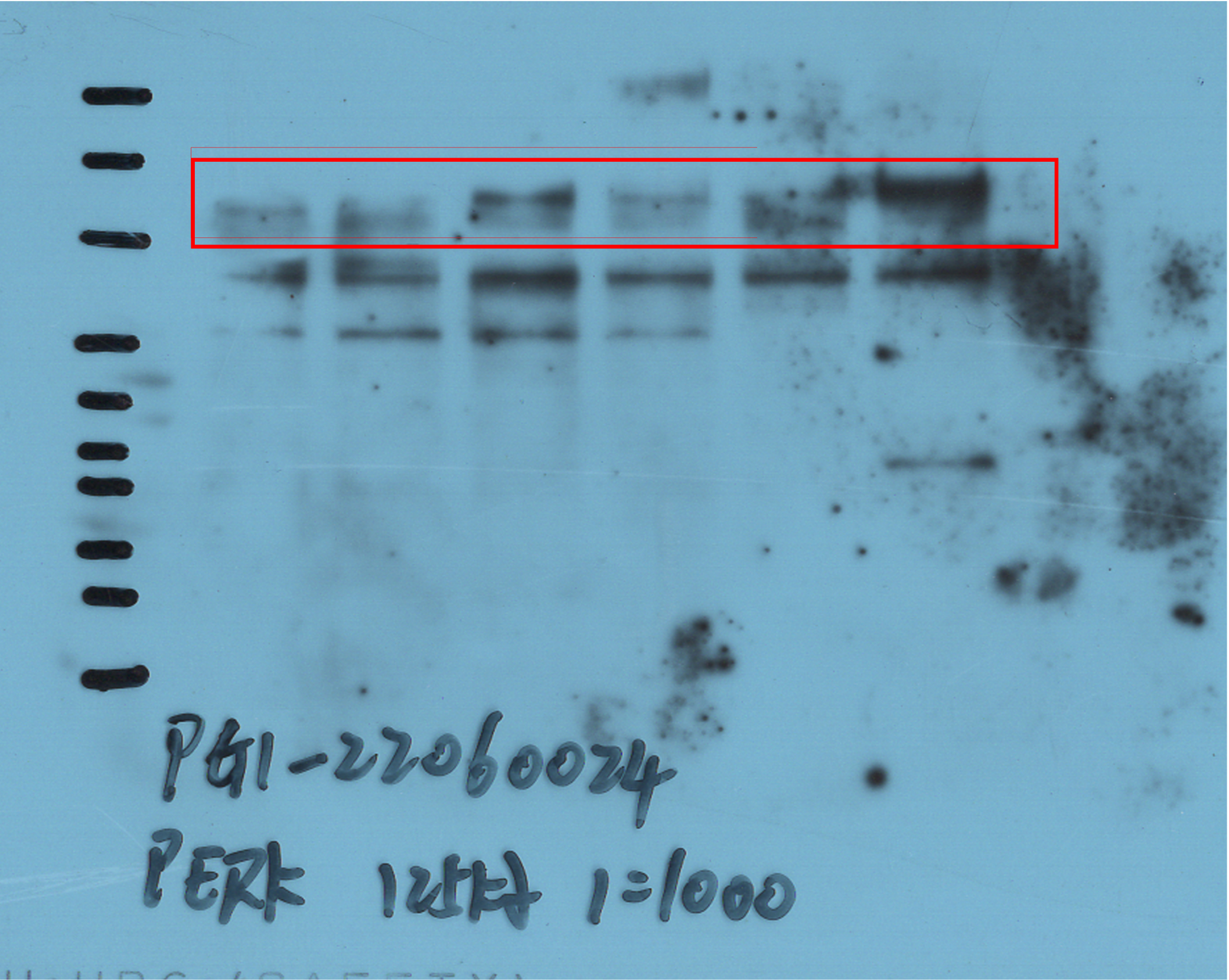

ATF4

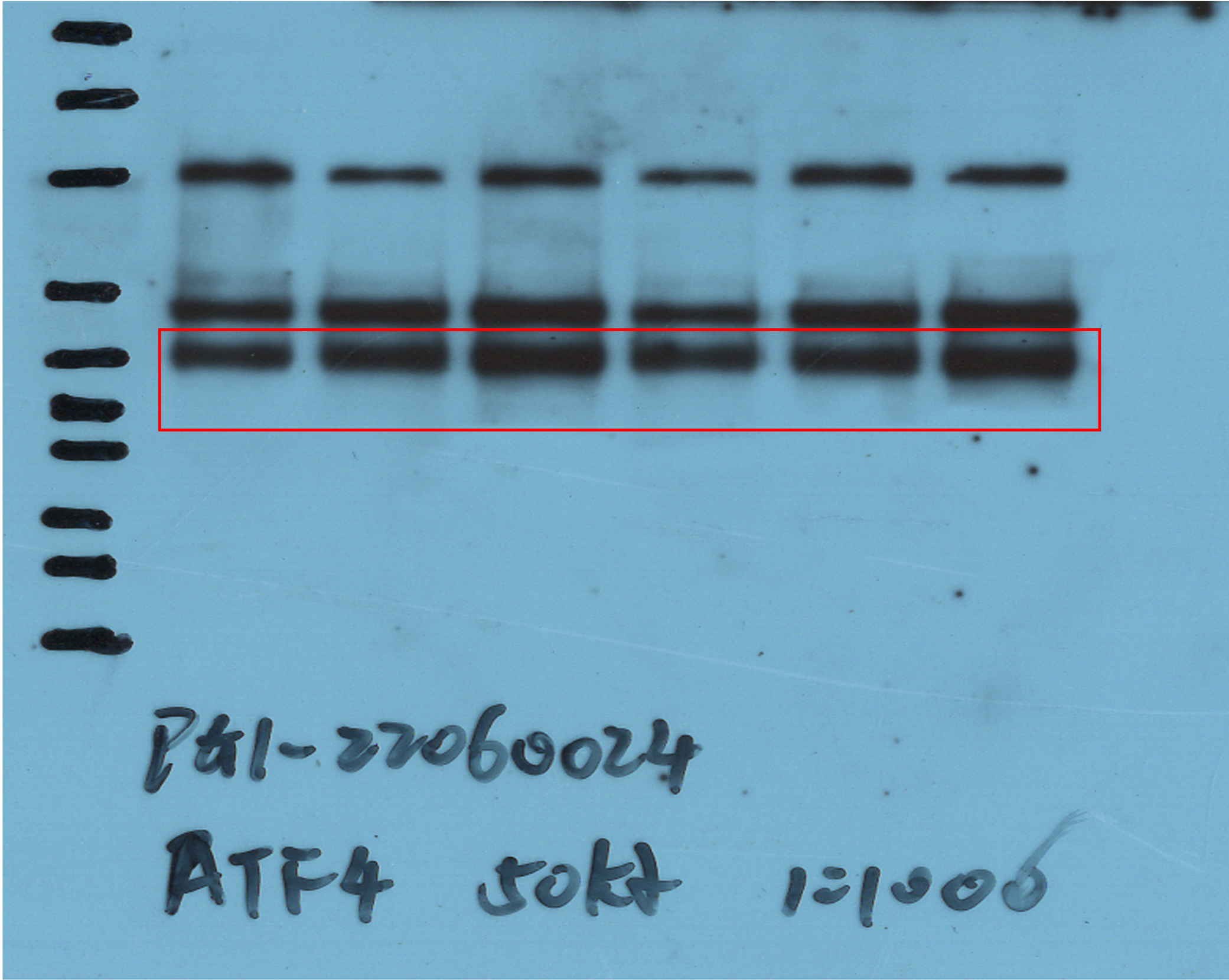

GAPDH

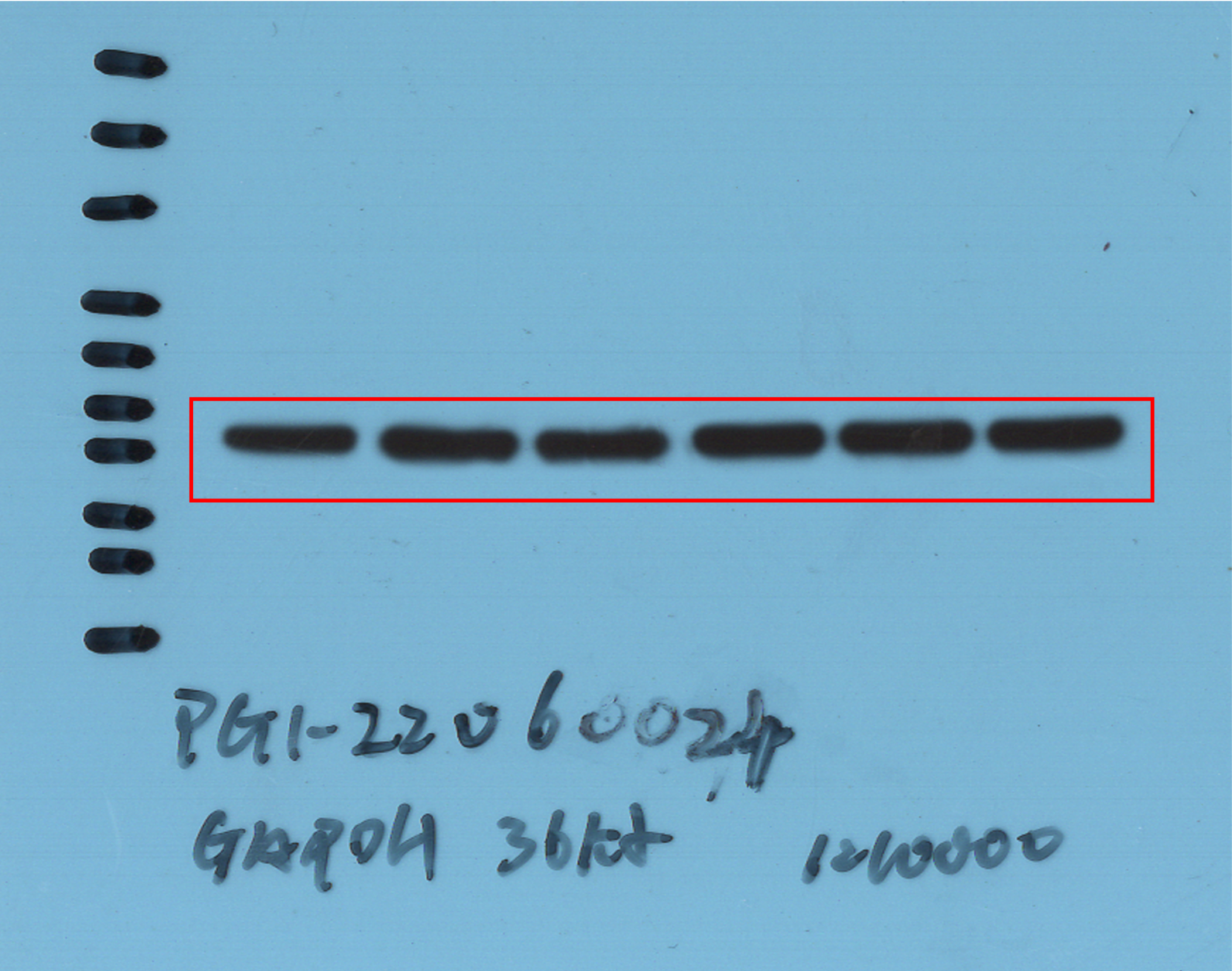

Supplement: Supplementary file 5 — Additional file 5. [file 12885_2024_11945_MOESM5_ESM.pdf]
