# Supplementary material for: Prognostic marker CD27 and its micro-environmental in multiple myeloma
Source: BMC Cancer. 2024 Mar 19;24:352. doi: 10.1186/s12885-024-11945-z (PMC10949675; doi:10.1186/s12885-024-11945-z)
Supplement: Supplementary file 3 — Additional file 3: Table S1. Designed primers for RT-qPCR. [file 12885_2024_11945_MOESM3_ESM.docx]

**Table S1** Designed primers for RT-qPCR

| Target | Forward | Reverse |
| --- | --- | --- |
| Human PERK | 5'-ATGACCGAAATGAGCTTCCTG-3' | 5'-GCTGGAGAACCCATGAGGT-3' |
| Human ATF4 | 5'-ACGATGAGACAGAGTTGCGAC-3' | 5'-ATCCAAGGCAGCAATTCTCCC-3' |
| Human GAPDH | 5'-ACAACAGCCTCAAGATCATCAGC-3' | 5'-GCCATCACGCCACAGTTTCC-3' |
