# Supplementary material for: Prognostic marker CD27 and its micro-environmental in multiple myeloma
Source: BMC Cancer. 2024 Mar 19;24:352. doi: 10.1186/s12885-024-11945-z (PMC10949675; doi:10.1186/s12885-024-11945-z)
Supplement: Supplementary file 4 — Additional file 4: Table S2. Basic clinical characteristics of 82 MM patients. [file 12885_2024_11945_MOESM4_ESM.docx]

**Table S2** Basic clinical characteristics of 82 MM patients.

| Variable | Median value or number |
| --- | --- |
| Male/Female | 45/37 |
| Age(year) | 63(47-83) |
| Hemoglobin(g/l) | 109(58-162) |
| Creatinine(μmol/l) | 126.74(43-611) |
| Calcium(mmol/l) | 2.49(1.74-4.10) |
| Albumin(g/l) | 36.07(15.90-54.00) |
| Lactate dehydrogenase (U/l) | 245.90(103-1085) |
| β2-MG (mg/l) | 5.59(1.30-14.54) |
| Proportion of bone marrow plasma cells (BMPC) (%) | 31.49(14.55-48.97) |
| M protein subtypes  IgG/IgA/IgD/light chain /non-secretory | 31/14/1/78/4 |
| ISS stage Ⅰ/Ⅱ/Ⅲ | 22/35/25 |
| Therapeutic schedule VRD/PCD/PAD | 37/27/18 |
